# Prognostic Models Using Machine Learning Algorithms and Treatment Outcomes of Occult Breast Cancer Patients

**DOI:** 10.3390/jcm12093097

**Published:** 2023-04-24

**Authors:** Jingkun Qu, Chaofan Li, Mengjie Liu, Yusheng Wang, Zeyao Feng, Jia Li, Weiwei Wang, Fei Wu, Shuqun Zhang, Xixi Zhao

**Affiliations:** 1 Department of Oncology, The Second Affiliated Hospital of Xi’an Jiaotong University, 157 West Fifth Street, Xi’an 710004, China; 2 Department of Otolaryngology, The Second Affiliated Hospital of Xi’an Jiaotong University, 157 West Fifth Street, Xi’an 710004, China; 3 Department of Radiation Oncology, The Second Affiliated Hospital of Xi’an Jiaotong University, 157 West Fifth Street, Xi’an 710004, China

**Keywords:** occult breast cancer, machine learning algorithm, prognosis, SEER, treatment

## Abstract

Background: Occult breast cancer (OBC) is an uncommon malignant tumor and the prognosis and treatment of OBC remain controversial. Currently, there exists no accurate prognostic clinical model for OBC, and the treatment outcomes of chemotherapy and surgery in its different molecular subtypes are still unknown. Methods: The SEER database provided the data used for this study’s analysis (2010–2019). To identify the prognostic variables for patients with ODC, we conducted Cox regression analysis and constructed prognostic models using six machine learning algorithms to predict overall survival (OS) of OBC patients. A series of validation methods, including calibration curve and area under the curve (AUC value) of receiver operating characteristic curve (ROC) were employed to validate the accuracy and reliability of the logistic regression (LR) models. The effectiveness of clinical application of the predictive models was validated using decision curve analysis (DCA). We also investigated the role of chemotherapy and surgery in OBC patients with different molecular subtypes, with the help of K-M survival analysis as well as propensity score matching, and these results were further validated by subgroup Cox analysis. Results: The LR models performed best, with high precision and applicability, and they were proved to predict the OS of OBC patients in the most accurate manner (test set: 1-year AUC = 0.851, 3-year AUC = 0.790 and 5-year survival AUC = 0.824). Interestingly, we found that the N1 and N2 stage OBC patients had more favorable prognosis than N0 stage patients, but the N3 stage was similar to the N0 stage (OS: N0 vs. N1, HR = 0.6602, 95%CI 0.4568–0.9542, *p* < 0.05; N0 vs. N2, HR = 0.4716, 95%CI 0.2351–0.9464, *p* < 0.05; N0 vs. N3, HR = 0.96, 95%CI 0.6176–1.5844, *p* = 0.96). Patients aged >80 and distant metastases were also independent prognostic factors for OBC. In terms of treatment, our multivariate Cox regression analysis discovered that surgery and radiotherapy were both independent protective variables for OBC patients, but chemotherapy was not. We also found that chemotherapy significantly improved both OS and breast cancer-specific survival (BCSS) only in the HR−/HER2+ molecular subtype (OS: HR = 0.15, 95%CI 0.037–0.57, *p* < 0.01; BCSS: HR = 0.027, 95%CI 0.027–0.81, *p* < 0.05). However, surgery could help only the HR−/HER2+ and HR+/HER2− subtypes improve prognosis. Conclusions: We analyzed the clinical features and prognostic factors of OBC patients; meanwhile, machine learning prognostic models with high precision and applicability were constructed to predict their overall survival. The treatment results in different molecular subtypes suggested that primary surgery might improve the survival of HR+/HER2− and HR−/HER2+ subtypes, however, only the HR−/HER2+ subtype could benefit from chemotherapy. The necessity of surgery and chemotherapy needs to be carefully considered for OBC patients with other subtypes.

## 1. Introduction

Occult breast cancer (OBC) is a rare malignant tumor, with an estimated incidence of 0.1% to 1% of all breast cancers. It is distinguished by the presence of pathological breast carcinoma in local lymph nodes or distal metastatic organs (usually axillary lymphadenopathy), but clinical or imaging examination fails to demonstrate the primary breast tumor [1,2,3]. Since OBC was initially described by Halsted in 1907 [4], its prognosis and management have been a matter of debate [1,5,6].

So far, the prognosis of OBC is still debatable. OBC patients have been shown to have a lower chance of mortality than non-OBC patients in some studies [7,8], whereas others have revealed comparable outcomes [9] or even significantly worse prognoses [10]. In addition, studies suggest that the prognosis factors of OBC patients vary greatly with different clinical features [3,5,11]. Moreover, nearly all these studies analyzed only patients with stage I to III, and so the effect of distant metastasis on OBC patients is still unclear. Therefore, there is an urgent need for prognostic prediction models to accurately answer the concerns of all OBC patients about survival and to help optimize their management.

So far, two studies have built several nomograms to predict the breast cancer specific survival (BCSS) of OBC patients [3,12]; however, one model can only be used for patients who have undergone surgery [3] and the other one can only be used for early-stage patients [12]. Moreover, the accuracy of the models is far from sufficient (AUC value or C-index is only around 0.7) and both of them did not assess the overall survival (OS). Consequently, a more widely available and accurate model is necessitated. Nowadays, with the advent of machine learning, analyzing the vast, multi-dimensional and multi-modal data generated by a clinical database has become easier [13,14]. Machine learning can also help us constructed artificial intelligence (AI) prognostic models, significantly improving their accuracy [14,15,16]. Our previous study successfully used a machine learning algorithm to predict the prognosis of breast cancer patients with initial bone metastases and greatly improve the accuracy [15]. However, no one has utilized machine learning to create prognostic models for OBC patients. Thus, we used six kinds of machine learning algorithm to create prognostic models and found that the LR algorithm performed best. Moreover, it has been challenging to conduct randomized controlled trials and standardize management because this particular type of breast cancer is extremely rare. A lot of retrospective studies focus on the effect of different treatment methods on the prognosis of all OBC patients [7,17,18,19,20,21], but no one has analyzed it in relation to different molecular subtypes, hence there is a need for a further inquiry.

Our study explores the factors influencing the prognosis of OBC patients using the most up-to-date Surveillance, Epidemiology and End Results (SEER) database and is the first one to create high-precision AI models to predict the 1, 3 and 5-year OS of OBC patients. We first investigated the role of the N0 stage, family income and months from diagnosis to therapy in OBC patients. Additionally, we further investigated the treatment outcomes of surgery and chemotherapy in different molecular subtypes, which have never been reported before, and found that primary surgery might improve the survival of OBC patients with HR+/HER2− and HR−/HER2+ subtypes; however, only the HR−/HER2+ subtype could benefit from chemotherapy. The necessity of surgery and chemotherapy needs to be carefully considered for OBC patients with other subtypes. This work gains insight into the prognosis of OBC patients and is helpful for their prognostic prediction and clinical management.

## 2. Materials and Methods

### 2.1. Data Source and Study Design

**Figure 1:**
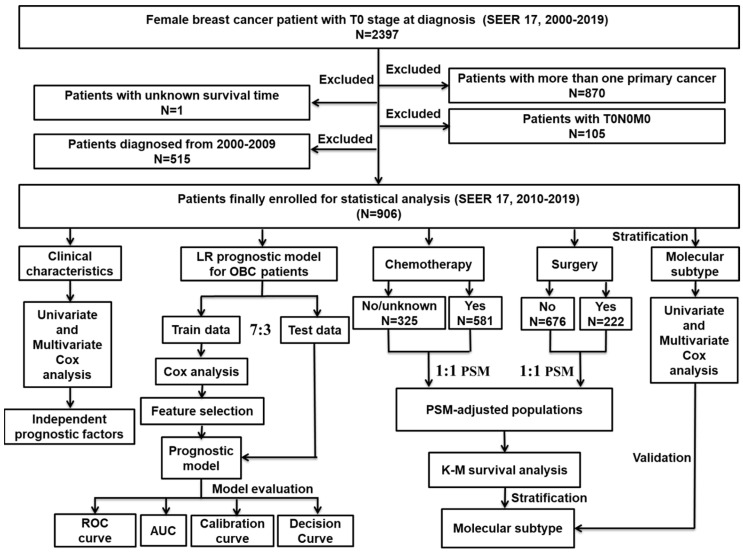
The flowchart details the procedure for carrying out the study and the analysis of data.

The workflow for the design and analyses of this study is illustrated in Figure 1. The data used for analysis in our study were obtained from the SEER database [SEER research plus data, 17 Regs, November 2021 Sub (2000–2019); version 8.4.0], which is openly accessible. As the information about distant metastasis and molecular subtypes were collected from 2010, and taking account of the data update, we only analyzed the data from 2010–2019. From this database, data on females with OBC were obtained. Inclusion criteria were as follows: (1) breast cancer proved to be the sole primary cancer the patient had been diagnosed with; (2) all these patients had shown histopathological and structural evidence consistent with the International Classification of Cancer Diseases Edition III (ICD-O-3); (3) aged ≥18 years; and (4) had T0 stage cancer according to the American Joint Committee on Cancer (AJCC). Exclusion criteria were as follows: (1) patients carried two or more primary cancers; (2) unexplainable TNM stage, such as T0N0M0; and (3) patients whose survival time was vague. Follow up was until the patient died, was lost to follow-up or until 31 December 2019.

### 2.2. Machine Learning Models

For feature selection, patients were sorted into train and test sets at random in a 7:3 ratio. In our train set, characteristics that were statistically significant in the multivariate Cox analysis, including age at diagnosis, molecular subtype, N stage, surgery, bone, lung, liver and brain metastases were included in our machine learning models to predict 1-, 3- and 5-year overall survival of OBC patients. Prior to excluding patients who were still alive but survived less than 1, 3 or 5 years at the follow-up cut-off date, the above analyses were conducted. A response variable for the survival information was obtained prior to launching the training program, with 1 denoting survival and 0 denoting death. On the test data, we compared the area under the curve (AUC value) of logistic regression (LR), random forest (RF), support vector machine (SVM), decision tree (ID3), k-Nearest Neighbor (kNN) and extreme gradient boosting (XGBoost). The LR model was further assessed by calibration curve and decision curve analysis.

### 2.3. LR

Logistic regression is known as log odds regression, it is a classification algorithm [22]. Under the assumption that the outcome variable has a probability distribution, logistic regression models the log odds of each patient experiencing the outcome linearly. This is converted to probabilities by means of a “sigmoid” function. Logistic regression is a highly interpretable algorithm and a hallmark of classical predictive modeling.

### 2.4. SVM

Support vector machines locate the hyperplanes that divide data points into two groups by mapping input vectors to higher dimensional feature spaces [23]. This maximizes the edge distance between the instance nearest to the boundary and the decision hyperplane. The identified hyperplane is the decision boundary between the two clusters and the resulting classifier has considerable generalization power.

### 2.5. ID3 and RF

The tree-structured classification method used by ID3, one of the earliest and most prevalent machine learning architectures, uses nodes to symbolize input factors and leaves to reflect decision outcomes [24]. Being based on the DT architecture, they are easy to interpret and fast to learn. Based on ID3, a random forest is generated by repetitively drawing k samples from the original training sample set, N, at random, followed by generating k classification trees according to the self-help sample set to generate the random forest.

### 2.6. XGBoost Model

The XGBoost algorithm modifies the gradient boosting algorithm by performing Taylor expansion of the loss function to the second order, adding a regularization term to the loss function, and solving for the extreme values of the loss function using Newton’s technique [25]. In addition, the technique employed in the XGBoost algorithm called “feature subsampling”, which can be understood as selecting a subset of all features to train each tree (similar to a random forest) in order to improve the generalization capability of the model, make it more diverse and prevent overfitting.

### 2.7. kNN

The kNN algorithm is founded on the premise that, if a sample falls under a category, most of the k closest to the neighboring samples in the feature space also fall under that category and share the same traits [26]. In determining the classification choice, the technique bases its determination of the category to which the sample to be classified corresponds solely on the category of the few most adjacent samples.

### 2.8. Statistical Analysis

To explore the connection between diverse pathological and clinical traits and patient survival rates, we applied univariate Cox regression models. To evaluate patient mortality risk and determine independent prognostic factors, further multivariate Cox analysis was carried out. Patients experiencing chemotherapy or surgical therapy and those receiving neither were paired on a 1:1 propensity score (PSM), according to variables in the univariate Cox regression, as a way to examine the role of these therapies on the outcome of patients with OBC [27]. On the PSM-adjusted population, we also conducted Kaplan–Meier (K-M) survival analysis [28] stratified by molecular subtype. Finally, we performed subgroup univariate, as well as multifactorial, Cox analyses in OBC patients according to molecular subtype. R software (version 4.0.2) was employed to conduct all the statistical analyses in this study. Statistical significance was determined to exist when the bilateral tail value was less than 0.05.

## 3. Results

### 3.1. Clinical Characteristics of OBC Patients

Eventually, we obtained information on 906 qualified OBC patients from the SEER database (2010 to 2019). The clinicopathological traits of OBC patients are displayed in Table 1 and summarized below. The patients’ median age was 62 years, of which 142 (15.67%) patients were younger than 50 years, and 92 (10.15%) patients were older than 80 years. In total, 449 (49.56%) patients began therapy immediately following diagnosis, whereas 377 (41.61%) patients began therapy after more than 1 month since diagnosis. For the molecular subtypes, HR+/HER2− made up 41.50%, followed by HR−/HER2− (16.11%), HR+/HER2+ (11.81%) and HR−/HER2+ (8.39%). In terms of ethnicity, 80.68% of the patients were white, and the most prevalent histopathological subtype was invasive ductal carcinoma (IDC; 30.57%). Regarding marital status, 49.89% of the patients were married and 14.79% were single. The proportions of stages N0 to N3 were 18.98%, 53.20%, 8.28% and 11.48%, respectively. Only 0.66% of the patients had grade I tumors, compared to 14.24% who developed grade III or IV. About 31.90% of the patients were found to have a decent annual family income of more than US$750,000. In the treatment field, only 24.50% of patients received surgery, 43.71% received radiotherapy and 64.13% received chemotherapy. Bone, lung, liver and brain metastases, respectively, accounted for 29.80%, 9.93%, 8.94% and 4.30% of all patients.

### 3.2. Univariate and Multivariate Cox Regression Analysis

To uncover significant factors influencing BCSS, as well as overall survival (OS) of OBC patients, we conducted univariate Cox regression analysis, including age at diagnosis, time from diagnosis to therapy, histological type, molecular subtype, marital status, N stage, race, grade, median family income (inflation-adjusted), distant metastases and information about treatment (Table 2).

Furthermore, we carried out multivariate Cox regression analysis to eliminate confounding factors and uncover independent factors correlated to BCSS and OS (Table 2). It showed that, in patients aged >80, distant metastases were significantly linked to inferior BCSS and OS. The HR−/HER2− subtype showed worse BCSS and OS compared with HR+/HER2− patients, whereas the HR+/HER2+ and HR−/HER2+ subtypes did not exhibit any difference. Patients at N1 and N2 stages had more favorable prognosis than at the N0 stage, but the N3 stage was similar to the N0 stage. In terms of treatment, only primary tumor surgery, and not chemotherapy or radiotherapy, could prolong both OS and BCSS according to multivariate Cox regression analysis, although radiotherapy could improve only the OS, just not the BCSS. Additionally, social variables such as family fiscal conditions and marriage status were analyzed; however, they are not independent prognosis factors for OBC.

### 3.3. Constructing and Assessing Predictive Models for the Estimation of OBC Patients’ Prognosis

In light of the above findings, patients were sorted into train and test data, at random, in a 7:3 ratio (Supplementary Table S1) and univariate and multivariate Cox analysis was used to analyze the train set again (Supplementary Table S2). Eight independent prognostic factors were chosen as model features, and prognostic models were created with six machine learning algorithms to assess the OS of OBC patients at 1, 3 and 5 years. For both train and test sets, we created predicted ROC curves and calculated their AUCs.

**Figure 2:**
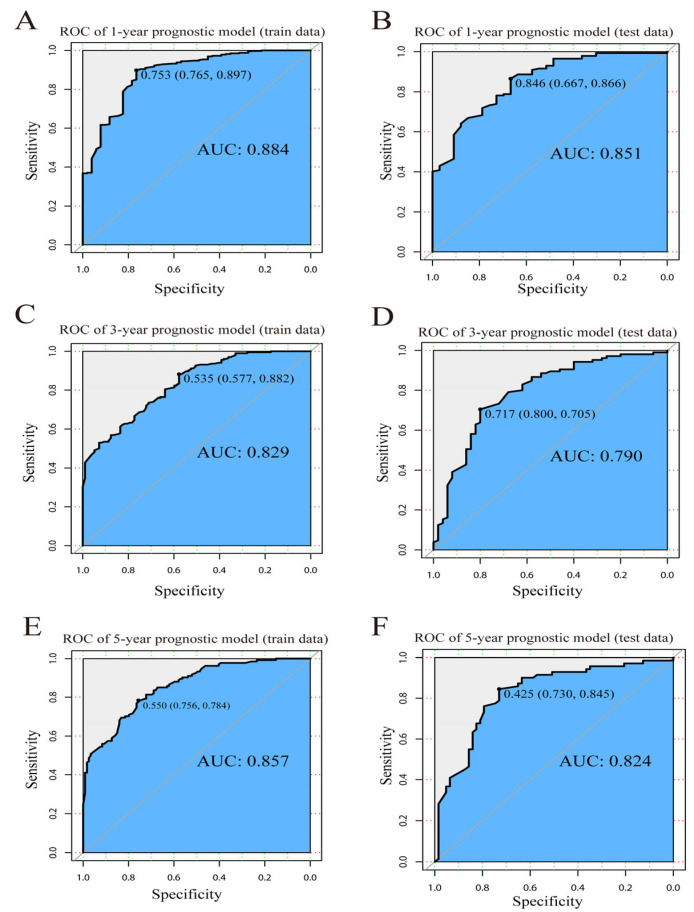
ROC curve of LR model. ROC curve for (**A**) the 1-year prognostic model using train data; (**B**) the 1-year prognostic model using test data; (**C**) the 3-year prognostic model using train data; (**D**) the 3-year prognostic model using test data; (**E**) the 5-year prognostic model using train data; and (**F**) the 5-year prognostic model using test data.

Our LR algorithm model manifested extraordinary efficiency in the prediction of OBC patient’ survival at 1 year (train set AUC = 0.884; test set AUC = 0.851), 3 years (train set AUC = 0.829; test set AUC = 0.790) and 5 years (train set AUC = 0.857; test set AUC = 0.824) (Figure 2A–F). In comparison with other machine learning algorithms, RF (1-year AUC = 0.818; 3-year AUC = 0.765; 5-year AUC = 0.824), XGBoost (1-year AUC = 0.795; 3-year AUC = 0.792; 5-year AUC = 0.829), ID3 (1-year AUC = 0.665; 3-year AUC = 0.755; 5-year AUC = 0.788), KNN (1-year AUC = 0.773; 3-year AUC = 0.711; 5-year AUC = 0.784) and SVM (1-year AUC = 0.550; 3-year AUC = 0.676; 5-year AUC = 0.766). The LR model performed best (Table 3).

**Figure 3:**
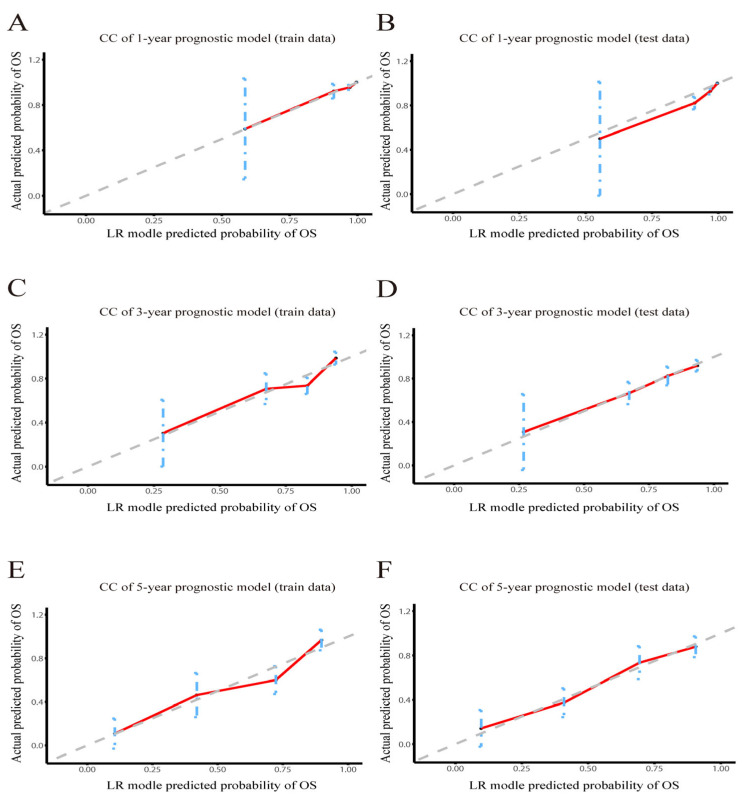
Calibration curve of LR model. Calibration curve of the LR model predicting OS in (**A**) the 1-year train set and (**B**) the test set; (**C**) the 3-year train set and (**D**) the test set; and (**E**) the 5-year train set and (**F**) the test set. CC: calibration curve.

**Figure 4:**
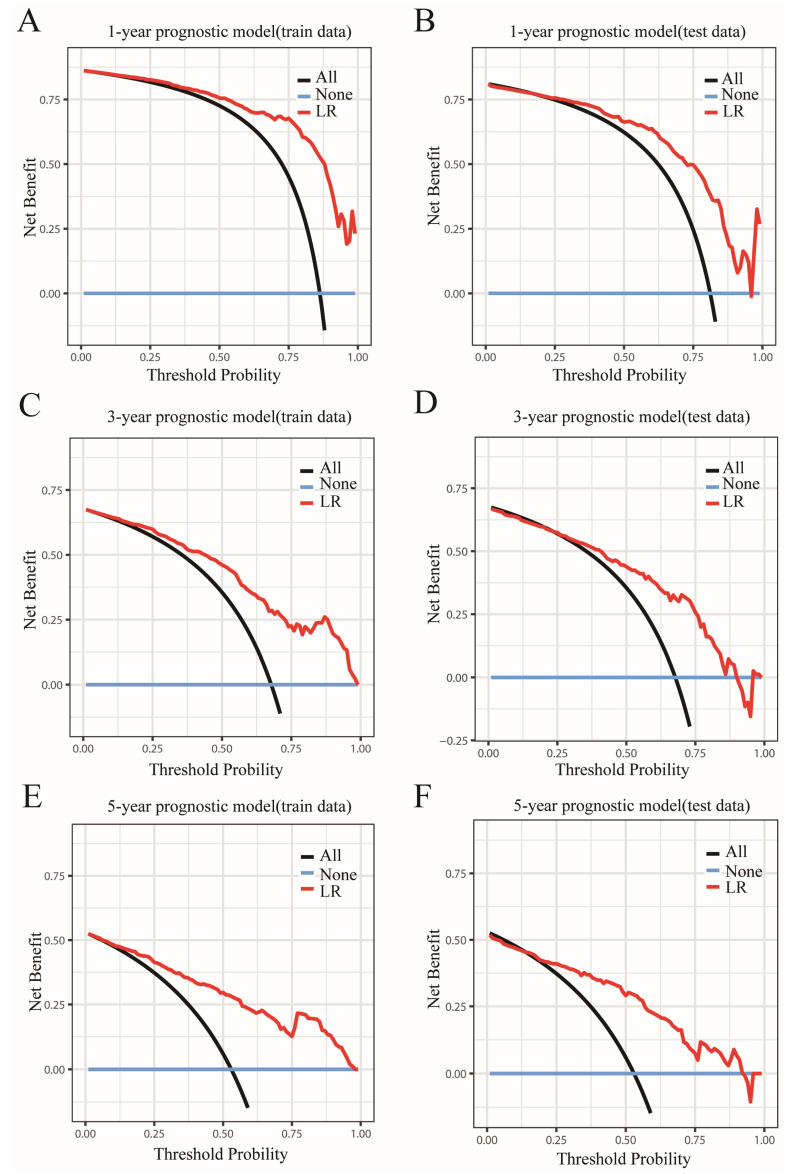
Decision curves of the LR model predicting OS. Decision curves of the LR model predicting OS in (**A**) the 1-year train set and (**B**) the test set; (**C**) the 3-year train set and (**D**) the test set; and (**E**) the 5-year train set and (**F**) the test set. The y-axis signifies the net benefit, and the x-axis signifies the threshold probability. The dark green line indicates that all of the patients are alive, and the blue line suggests that none of the patients are alive.

Then, the accuracy of our LR models was further assessed using calibration curves [29]. According to the calibration curves of the train and test sets (Figure 3A–F), the predicted values of LR models were perfectly in keeping with the observed values, indicating that LR models had remarkable accuracy. After determining the accuracy of the prediction models, we further analyzed their clinical applicability via decision curve analysis (DCA) [30]. The results showed that the LR models had a wide threshold probability range and a good net benefit in predicting 1-year, 3-year and 5-year OS rates for OBC (Figure 4A–F). Overall, our models performed well.

### 3.4. Benefits of Chemotherapy in OBC Patients Subdivided by Molecular Subtype

Unexpectedly, chemotherapy was not an independent prognostic factor for OBC patients in our multivariate Cox regression analysis (Table 2). Hence, we took a further look at how chemotherapy affected OBC patient prognosis. We contrasted the baseline features of patients receiving chemotherapy with those without chemotherapy (Table 4). These two groups had different baselines. Therefore, the observed disparity was adjusted with the help of propensity score matching (PSM). After PSM adjustment, there existed no discernible differences in baseline characteristics (Table 4).

**Figure 5:**
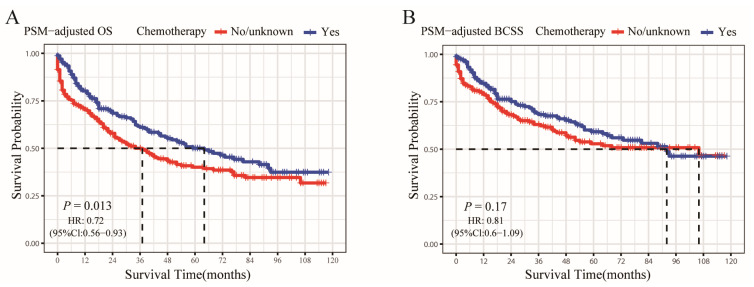
OS and BCSS of chemotherapy-treated OBC patients after PSM adjustment. Kaplan–Meier (K-M) survival analysis: (**A**) PSM-adjusted OS of chemotherapy-treated OBC; (**B**) PSM-adjusted BCSS of chemotherapy-treated OBC.

**Figure 6:**
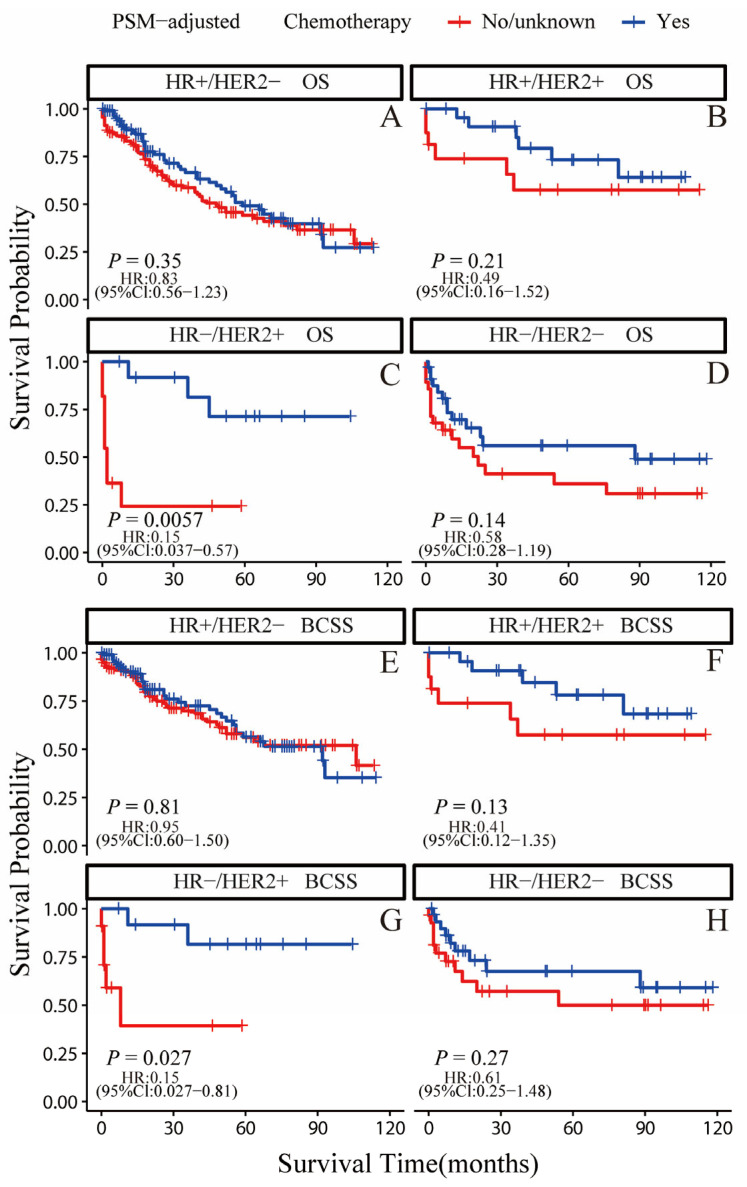
OS and BCSS of chemotherapy-treated OBC patients after PSM adjustment (stratified by molecular subtype). (**A**) OS of HR+/HER2− subtype; (**B**) OS of HR+/HER2+ subtype; (**C**) OS of HR−/HER2+ subtype; (**D**) OS of HR−/HER2− subtype; (**E**) BCSS of HR+/HER2− subtype; (**F**) BCSS of HR+/HER2+ subtype; (**G**) BCSS of HR−/HER2+ subtype; (**H**) BCSS of HR−/HER2− subtype.

According to the PSM-adjusted data, the chemotherapy group’s overall risk of death was reduced by about 28% (*p* = 0.013, HR: 0.72; 95% CI: 0.56–0.93) (Figure 5A), whereas there was no difference in the risk of breast cancer-related death (*p* = 0.17, HR: 0.81; 95% CI: 0.6–1.09) (Figure 5B). Only the HR−/HER2+ subgroup could substantially benefit from chemotherapy in terms of OS and BCSS, according to the stratified K-M survival study (Figure 6C,G); however, it did not show any benefit for the OS and BCSS of other three subtypes (Figure 6A,B,D–F,H). To further validate these results, we divided all the 906 eligible OBC patients into four groups, according to molecular subtype, and performed univariate and multivariate Cox analyses again (Supplementary Table S3). It showed that only the HR−/HER2+ subtype could benefit from chemotherapy, which is consistent with our results for the PSM-adjusted K-M survival analysis.

### 3.5. Benefits of Surgery for OBC Patients Subdivided by Molecular Subtype

In view of the above results, we looked further into the influence of surgery on the prognosis of OBC patients with distinctive subtypes. Using the same PSM method, there appeared no significant differences between patients receiving surgical treatment and those without surgery in terms of baseline characteristics (Table 5).

**Figure 7:**
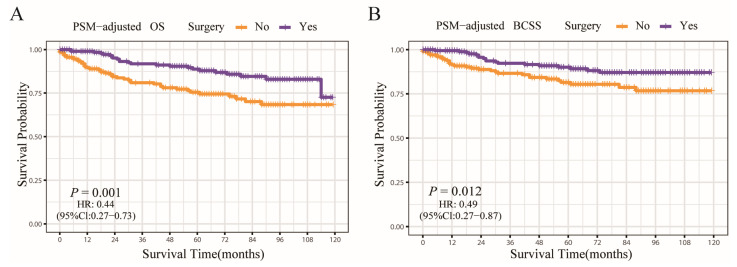
OS and BCSS of surgery-treated OBC patients after PSM adjustment. (**A**) OS of surgery-treated OBC patients after PSM adjustment; (**B**) BCSS of surgery-treated OBC patients after PSM adjustment.

**Figure 8:**
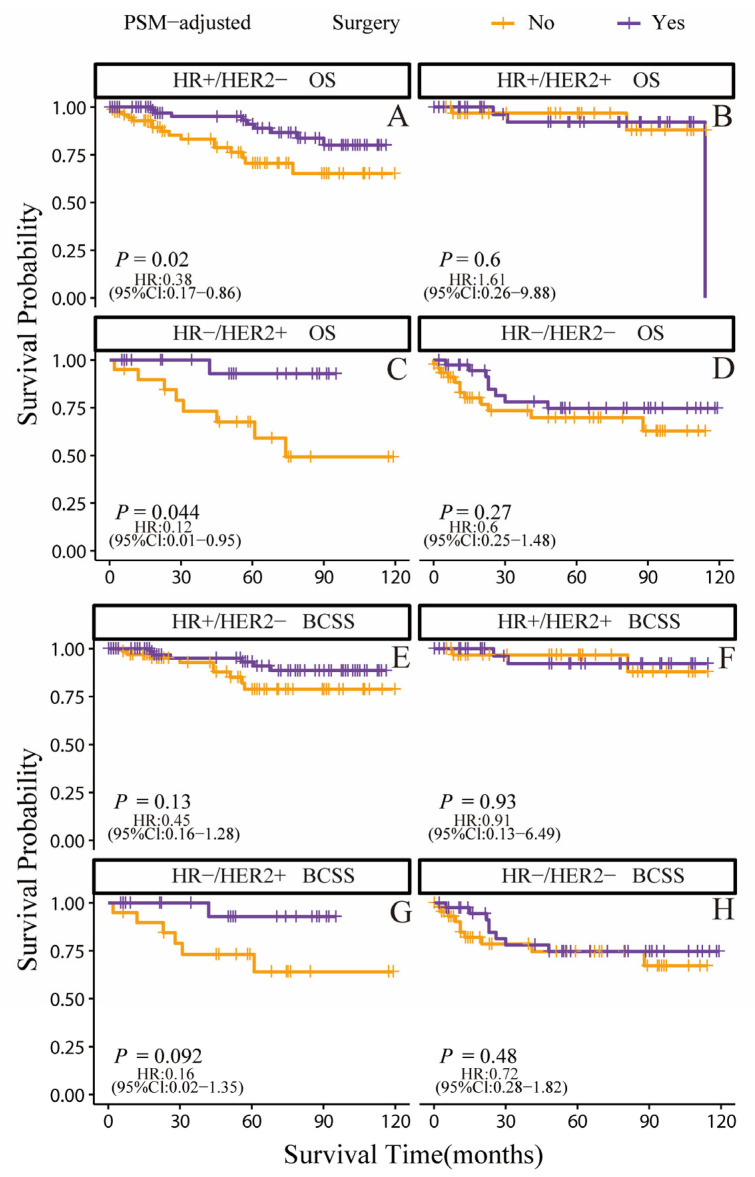
OS and BCSS of surgery-treated OBC patients after PSM adjustment. (stratified by molecular subtype). (**A**) OS of HR+/HER2− subtype; (**B**) OS of HR+/HER2+ subtype; (**C**) OS of HR−/HER2+ subtype; (**D**) OS of HR−/HER2− subtype; (**E**) BCSS of HR+/HER2− subtype; (**F**) BCSS of HR+/HER2+ subtype; (**G**) BCSS of HR−/HER2+ subtype; (**H**) BCSS of HR−/HER2− subtype.

According to the PSM-adjusted data, the surgery group’s overall risk of death was reduced by around 56% (*p* = 0.001, HR: 0.44; 95% CI: 0.27–0.73) (Figure 7A), with the risk of breast cancer-related death reduced by approximately 51% (*p* = 0.012, HR: 0.49; 95% CI: 0.27–0.87) (Figure 7B). The stratified K-M survival analysis uncovered that surgical treatment significantly improved OS in the HR+/HER2− and HR−/HER2+ subtypes (Figure 8A,C). However, there was no significant difference in HR+/HER2+ and HR−/HER2− subtypes (Figure 8B,D). In addition, the effect of surgical treatment on BCSS in patients with all subtypes was similar (Figure 8E–H). To further validate these results, we divided all the 906 eligible OBC patients into four groups, according to molecular subtype, and performed univariate and multivariate Cox analyses again (Supplementary Table S3). It showed that surgical intervention was proven to be an independent prognostic factor only for HR+/HER2− and HR−/HER2+ subtypes, supporting our findings from the PSM-adjusted K-M survival analysis.

## 4. Discussion

OBC is an unusual clinical entity and represents a therapeutic challenge for doctors [31]. Since this type of breast cancer is quite rare, its prognosis remains debatable, and standardized management of OBC is still difficult [1,6]. Some large-sample retrospective studies using SEER could help solve the problem of rare cases, but most such cases in previous studies have a large time span [7,20,21] and some cases that were not OBC might have been considered so in the past because of the limitations of imaging technology [6,32,33]. The present study, as far as we are aware, is the most up-to-date one to examine the clinical traits and prognosis of OBC patients. In two recent investigations, several nomogram prediction models for OBC patients were created using SEER populations [3,12]; however, their models could not predict OS and could only be used for patients who had undergone surgery [3] or were at an early-stage [12]. Moreover, the accuracy of their models is far from sufficient. Thus, our research is also the first to develop AI prognostic models for OBC patients, and our LR models are the most widely available and are more accurate in predicting the OS of OBC patients.

This study identified several independent factors associated with poor prognosis, including age ≥80, triple negative molecular subtype, N0/N3 stage, and distant metastasis. Some studies have shown that OBC patients aged ≥70 are more likely to develop worse OS [20,21], whereas other studies have claimed that age was not a risk factor [3,5,12]. We looked at a wider range of age categories and discovered a worse OS for people aged ≥80. Compared to the HR+/HER2− subtype, only the HR−/HER2− subtype showed poorer survival and some studies also showed that ER+ was an independent favorable factor [3,5], which implies the importance of endocrine therapy for HR+ OBC patients. On the contrary, several studies have indicated that OBC patients of different subtypes showed no difference in terms of survival [7,12,20], which could be attributed to the diverse enrolled populations. Interestingly, compared with N0 stage, the OBC patients at N1 and N2 stages showed better OS and BCSS, but there was no difference between the N0 and N3 stages. Perhaps the prime reason for this is that N0 stage OBC must be accompanied by distant metastasis, coupled with the fact that distant metastasis is also an unfavorable independent prognostic factor; thus, OBC patients at N0 and N3 stages had the worst prognosis. Some previous studies have shown that N2+ is an unfavorable independent prognostic factor of OBC [3,12], but all their references were at the N1 stage; in other words, we are the first to have investigated the role of the N0 stage in OBC patients. We also detected the role of family income and months from diagnosis to therapy, which have never been reported in OBC patients; although both of these are not prognosis factors in OBC.

In terms of treatment, surgery and radiotherapy were both independent protective variables for OBC patients, according to our multivariate Cox regression analysis of the data, whereas chemotherapy was not. Many studies have focused on the therapeutic effects of different surgical methods, such as mastectomy or breast-conserving treatment, combined with radiotherapy and indicated that breast conservation can be considered in patients with OBC [17,19,20,21,34,35]. Surprisingly, previous studies have also reported that chemotherapy was not an independent prognostic factor in OBC patients [3,11,20]. However, no one had investigated the role of chemotherapy and surgery in OBC patients with different subtypes, thus, we further explored this issue. We found that chemotherapy significantly improved both OS and BCSS only in OBC patients with the HR−/HER2+ subtype, suggesting that anti-HER2-targeted therapy combined with chemotherapy may prolong the survival of OBC patients and that endocrinotherapy is more important in the HR+ subtype than chemotherapy. We also found that surgery appeared to be an independent prognostic factor only when it comes to HR+/HER2− and HR−/HER2+ subtypes, indicating that comprehensive endocrinotherapy and surgical treatment is very important for the HR+/HER2− subtype and that multimodal treatment, involving chemotherapy, surgical treatment and anti-HER2-targeted therapy, could benefit the HR−/HER2+ subtype. For OBC patients with other subtypes, the necessity of surgery and chemotherapy needs to be carefully considered.

Our study may have several limitations despite its promising discoveries. First, for systemic therapy, there is no detailed information on, for example, the dosage of each drug or the chemotherapy formula, in current database; hence, we were unable to find out more about the relationships between various chemotherapy regimens and the survival of patients. Meanwhile, the most recent version of the SEER database does not contain any information about endocrine therapy. Second, the SEER database represents the general situation well, but due to ethnic differences, it may not always apply to Asian, and especially Chinese, patients. Third, owing to the limited number of cases, the number of matches in PSM was not 100%; so selection bias might have occurred.

## 5. Conclusions

We analyzed the clinical features of OBC patients and constructed three high-precision and applicability machine-learning prognostic models to predict their survival. According to our analysis of possible prognostic variables for OBC patients, the survival of OBC patients with the HR−/HER2+ subgroup may benefit from chemotherapy, whereas the prognosis for the HR+/HER2− and HR−/HER2+ subtypes may be benefited by primary surgery.

## Figures and Tables

**Table 1:** Baseline characteristics of patients with occult breast cancer (OBC).

Characteristic		Cases	%
**Age at diagnosis**	<50	142	15.67%
	50–59	247	27.26%
	60–69	252	27.81%
	70–79	173	19.09%
	80+	92	10.15%
**Months from diagnosis to therapy**	0 month	449	49.56%
	≥1 month	377	41.61%
	unknown	80	8.83%
**Subtype**	HR+/HER2−	376	41.50%
	HR+/HER2+	107	11.81%
	HR−/HER2+	76	8.39%
	HR−/HER2−	146	16.11%
	unknown	201	22.19%
**Race**	white	731	80.68%
	black	95	10.49%
	other	74	8.17%
	unknown	6	0.66%
**Histological type**	IDC	277	30.57%
	ILC	78	8.61%
	other	551	60.82%
**Marital status**	married	452	49.89%
	singled	134	14.79%
	divorced/other	145	16.00%
	widowed	145	16.00%
	unknown	30	3.31%
**N stage**	N0	172	18.98%
	N1	482	53.20%
	N2	75	8.28%
	N3	104	11.48%
	unknown	73	8.06%
**Grade**	I; well differentiated	6	0.66%
	II; moderate differentiated	28	3.09%
	III/IV; poorly differentiated	129	14.24%
	unknown	743	82.01%
**Median household income (inflation ajusted)**	<44,999$	73	8.06%
	45,000–54,999$	121	13.36%
	55,000–64,999$	215	23.73%
	65,000–74,999$	208	22.96%
	75,000$+	289	31.90%
**Chemotherapy**	no/unknown	325	35.87%
	yes	581	64.13%
**Radiotherapy**	no/unknown	510	56.29%
	yes	396	43.71%
**Surgery**	no	676	74.61%
	yes	222	24.50%
	unknown	8	0.88%
**Bone metastases**	no	612	67.55%
	yes	270	29.80%
	unknown	24	2.65%
**Liver metastases**	no	798	88.08%
	yes	81	8.94%
	unknown	27	2.98%
**Lung metastases**	no	789	87.09%
	yes	90	9.93%
	unknown	27	2.98%
**Brain metastases**	no	838	92.49%
	yes	39	4.30%
	unknown	29	3.20%

**Table 2:** Univariate and multivariate Cox analysis of OBC characteristics.

	Univariate Cox Analysis						Multivariate Cox Analysis					
	OS			BCSS			OS			BCSS		
	HR	95%CI	*p* Value	HR	95%CI	*p* Value	HR	95%CI	*p* Value	HR	95%CI	*p* Value
**Age at dignosis**												
<50	reference			reference			reference			reference		
50–59	1.338	0.9089–1.970	0.14	1.439	0.9340–2.216	0.099	0.79	0.4593–1.3585	0.39	0.7589	0.4224–1.3634	0.36
60–69	1.744	1.1980–2.538	**	1.474	0.9554–2.275	0.079	1.1716	0.6870–1.9979	0.56	0.9772	0.5434–1.7572	0.94
70–79	2.195	1.4956–3.221	***	2.015	1.2990–3.125	**	1.3483	0.7734–2.3508	0.29	1.2467	0.6879–2.2593	0.47
80+	4.59	3.0608–6.882	***	3.645	2.2560–5.888	***	2.1283	1.0377–4.3651	*	1.5213	0.7263–3.1868	0.27
**Months from diagnosis to therapy**												
0 month	reference			reference			/	/	/	/	/	/
≥1 month	1.085	0.8694–1.353	0.47	1.075	0.8335–1.388	0.57	/	/	/	/	/	/
**Subtypes**												
HR+/HER2−	reference			reference			reference			reference		
HR+/HER2+	0.5526	0.3583–0.8523	**	0.6496	0.4018–1.050	0.078	0.8763	0.5335–1.4395	0.60	1.0001	0.5760–1.7363	1.0
HR−/HER2+	0.8551	0.5586–1.3088	0.47	0.9111	0.5578–1.488	0.71	0.8652	0.4985–1.5016	0.61	0.9611	0.5259–1.7564	0.90
HR−/HER2−	1.1147	0.8188–1.5177	0.49	1.2257	0.8594–1.748	0.26	1.9999	1.3739–2.9111	***	2.6362	1.7191- 4.0426	***
**Race**												
white	reference			reference			/	/	/	/	/	/
black	0.8584	0.6055–1.217	0.39	0.738	0.4767–1.143	0.17	/	/	/	/	/	/
other	0.8479	0.5642–1.274	0.43	0.8337	0.5161–1.347	0.46	/	/	/	/	/	/
**Histological type**												
IDC	reference			reference			reference			reference		
ILC	2.951	2.036–4.276	***	3.267	2.178–4.899	***	1.5221	0.8794–2.6345	0.13	2.0359	1.1437–3.6242	*
other	1.875	1.434–2.452	***	1.586	1.167–2.156	**	0.8826	0.6165–1.2634	0.49	0.7979	0.5335–1.1933	0.27
**Marriage status**												
married	reference			reference			reference			/	/	/
divorced/separated/other	1.191	0.8917–1.591	0.24	1.105	0.7873–1.551	0.56	1.1943	0.8067–1.7680	0.38	/	/	/
single	1.272	0.9457–1.711	0.11	1.126	0.7898–1.605	0.51	1.0995	0.7145–1.6920	0.67	/	/	/
widowed	1.522	1.1612–1.996	**	1.281	0.9222–1.779	0.14	0.7685	0.4741–1.2456	0.67	/	/	/
widowed/divorced/other												
**N Stage**												
N0	reference			reference			reference			reference		
N1	0.3618	0.28350–0.4616	***	0.3474	0.26222–0.4604	***	0.6602	0.4568–0.9542	*	0.5671	0.3734–0.8612	**
N2	0.1695	0.09877–0.2907	***	0.1321	0.06642–0.2628	***	0.4716	0.2351–0.9464	*	0.3411	0.1456–0.7991	*
N3	0.3757	0.25815–0.5468	***	0.4095	0.27027–0.6206	***	0.9892	0.6176–1.5844	0.96	1.0411	0.6160–1.7595	0.88
**Grade**												
well differentiated	reference			reference			/	/	/	/	/	/
moderately differentiated	2.73	0.3518–21.18	0.34	/	/	/	/	/	/	/	/	/
poorly differentiated	2.271	0.3118–16.54	0.42	/	/	/	/	/	/	/	/	/
**Median household income (inflation ajusted)**												
<45,000$	reference			reference			/	/	/	/	/	/
45,000–54,999$	0.9608	0.6266–1.473	0.85	0.8186	0.4983–1.345	0.43	/	/	/	/	/	/
55,000–64,999$	0.8483	0.5717–1.259	0.41	0.7758	0.4949–1.216	0.27	/	/	/	/	/	/
65,000–74,999$	0.701	0.4634–1.060	0.093	0.6992	0.4385–1.115	0.13	/	/	/	/	/	/
>74,999$	0.8262	0.5619–1.215	0.33	0.714	0.4592–1.110	0.14	/	/	/	/	/	/
**Chemotherapy**												
no/unknown	reference			reference			reference			reference		
yes	0.3753	0.3065–0.4594	***	0.447	0.3525–0.567	***	0.7487	0.5273–1.0632	0.11	0.8836	0.5923–1.3181	0.54
**Radiotherapy**												
no/unknown	reference			reference			reference			reference		
yes	0.4247	0.3397–0.5308	***	0.4806	0.3719–0.621	***	0.6773	0.4939–0.9289	*	0.8091	0.5692–1.1503	0.24
**Surgery**												
no/unknown	reference			reference			reference			reference		
yes	0.1427	0.09428–0.216	***	0.1583	0.09917–0.2526	***	0.4271	0.2609–0.6990	***	0.4491	0.2593–0.7777	**
**Bone metastasis**												
no/unknown	reference						reference					
yes	3.568	2.892–4.402	***	4.121	3.216–5.281	***	2.0779	1.4807–2.9161	***	2.5002	1.6968–3.6839	***
**Liver metastasis**												
no/unknown	reference			reference			reference			reference		
yes	4.443	3.358–5.877	***	4.553	3.279–6.322	***	2.4709	1.5316–3.9863	***	2.604	1.5423–4.3963	***
**Lung metastasis**												
no/unknown	reference			reference			reference			reference		
yes	4.03	3.106–5.229	***	3.432	2.483–4.745	***	2.572	1.7343–3.8142	***	2.4935	1.5642–3.9748	***
**Brain metastasis**												
no/unknown	reference			reference			reference			reference		
yes	2.83	1.933–4.142	***	2.876	1.838- 4.5	***	2.3986	1.3387–4.2976	**	2.0765	1.0757–4.0083	*

* *p* < 0.05, ** *p* < 0.01, *** *p* < 0.001.

**Table 3:** Performance of prognostic models constructed by machine learning algorithms using test data (area under the ROC curve).

	1-Year Survival	3-Year Survival	5-Year Survival
**LR**	0.851	0.790	0.824
**RF**	0.818	0.765	0.824
**XGBoost**	0.795	0.792	0.829
**ID3**	0.665	0.755	0.788
**KNN**	0.773	0.711	0.784
**SVM**	0.550	0.676	0.766

**Table 4:** Comparison of patient features according to chemotherapy before and after propensity score matching (PSM).

Characteristics	Unmatched Cohort					1:1 Propensity Score Matched (PSM) Cohort				
	Chemotherapy Not Given		Chemotherapy		Unadjusted	Chemotherapy Not Given		Chemotherapy		PSM-Adjusted
	N = 325	%	N = 581	%	*p* Value	N = 234	%	N = 234	%	*p* Value
**Age at diagnosis**					***					0.462
<50	22	6.77%	120	20.65%		21	8.97%	31	13.25%	
50–59	68	20.92%	179	30.81%		57	24.36%	64	27.35%	
60–69	82	25.23%	170	29.26%		71	30.34%	67	28.63%	
70–79	85	26.15%	88	15.15%		56	23.93%	49	20.94%	
80+	68	20.92%	24	4.13%		29	12.39%	23	9.83%	
**Subtype**					***					0.586
HR+/HER2−	149	45.85%	227	39.07%		114	48.72%	104	44.44%	
HR+/HER2+	16	4.92%	91	15.66%		16	6.84%	24	10.26%	
HR−/HER2+	11	3.38%	65	11.19%		11	4.70%	13	5.56%	
HR−/HER2−	33	10.15%	113	19.45%		28	11.97%	33	14.10%	
unknown	116	35.69%	85	14.63%		65	27.78%	60	25.64%	
**Race**					*					0.778
white	277	85.23%	454	78.14%		199	85.04%	191	81.62%	
black	25	7.69%	70	12.05%		19	8.12%	22	9.40%	
other	20	6.15%	54	9.29%		15	6.41%	20	8.55%	
unknown	3	0.92%	3	0.52%		1	0.43%	1	0.43%	
**Histological type**					***					0.668
IDC	64	19.69%	213	36.66%		51	21.79%	57	24.36%	
ILC	37	11.38%	41	7.06%		29	12.39%	24	10.26%	
other	224	68.92%	327	56.28%		154	65.81%	153	65.38%	
**Marital status**					***					0.893
married	129	39.69%	323	55.59%		104	44.44%	107	45.73%	
divorced/separated/other	52	16.00%	93	16.01%		40	17.09%	44	18.80%	
single	51	15.69%	83	14.29%		35	14.96%	37	15.81%	
widowed	79	24.31%	66	11.36%		46	19.66%	38	16.24%	
unknown	14	4.31%	16	2.75%		9	3.85%	8	3.42%	
**N stage**					***					0.099
N0	111	34.15%	61	10.50%		59	25.21%	49	20.94%	
N1	131	40.31%	351	60.41%		115	49.15%	119	50.85%	
N2	9	2.77%	66	11.36%		9	3.85%	23	9.83%	
N3	27	8.31%	77	13.25%		25	10.68%	21	8.97%	
unknown	47	14.46%	26	4.48%		26	11.11%	22	9.40%	
**Grade**					***					0.076
well	0	0.00%	6	1.03%		0	0.00%	4	1.71%	
moderately	3	0.92%	25	4.30%		3	1.28%	6	2.56%	
poorly	21	6.46%	108	18.59%		20	8.55%	28	11.97%	
unknown	301	92.62%	442	76.08%		211	90.17%	196	83.76%	
**Median household income (inflation adjusted)**					0.133					0.532
<45,000$	32	9.85%	41	7.06%		24	10.26%	16	6.84%	
45,000–54,999$	44	13.54%	77	13.25%		31	13.25%	30	12.82%	
55,000–64,999$	86	26.46%	129	22.20%		64	27.35%	59	25.21%	
65,000–74,999$	62	19.08%	146	25.13%		42	17.95%	53	22.65%	
>74,999$	101	31.08%	188	32.36%		73	31.20%	76	32.48%	
**Surgery**					***					0.254
no	295	90.77%	381	65.58%		209	89.32%	197	84.19%	
yes	24	7.38%	198	34.08%		24	10.26%	35	14.96%	
unknown	6	1.85%	2	0.34%		1	0.43%	2	0.85%	
Radiotherapy					***					0.556
no/unknown	245	75.38%	265	45.61%		160	68.38%	153	65.38%	
yes	80	24.62%	316	54.39%		74	31.62%	81	34.62%	
**Bone metastases**					***					1
no	160	49.23%	452	77.80%		137	58.55%	137	58.55%	
yes	149	45.85%	121	20.83%		90	38.46%	90	38.46%	
unknown	16	4.92%	8	1.38%		7	2.99%	7	2.99%	
**Liver metastases**					***					0.908
no	273	84.00%	525	90.36%		202	86.32%	199	85.04%	
yes	33	10.15%	48	8.26%		25	10.68%	28	11.97%	
unknown	19	5.85%	8	1.38%		7	2.99%	7	2.99%	
**Lung metastases**					***					0.876
no	255	78.46%	534	91.91%		196	83.76%	200	85.47%	
yes	52	16.00%	38	6.54%		30	12.82%	27	11.54%	
unknown	18	5.54%	9	1.55%		8	3.42%	7	2.99%	
**Brain metastases**					*					0.979
no	293	90.15%	545	93.80%		214	91.45%	214	91.45%	
yes	14	4.31%	25	4.30%		12	5.13%	13	5.56%	
unknown	18	5.54%	11	1.89%		8	3.42%	8	3.42%	

* *p* < 0.05, *** *p* < 0.001.

**Table 5:** Comparison of patient characteristics according to surgical treatment before and after propensity score matching (PSM).

Characteristics	Unmatched Cohort					1:1 Propensity Score Matched (PSM) Cohort				
	Surgery Not Given		Surgery		Unadjusted	Surgery Not Given		Surgery		PSM-Adjusted
	N = 676	%	N = 222	%	*p* Value	N = 209	%	N = 209	%	*p* Value
**Age at diagnosis**					***					0.089
<50	74	10.95%	67	30.18%		36	17.22%	56	26.79%	
50–59	174	25.74%	71	31.98%		77	36.84%	69	33.01%	
60–69	191	28.25%	57	25.68%		70	33.49%	57	27.27%	
70–79	149	22.04%	23	10.36%		18	8.61%	23	11.00%	
80+	88	13.02%	4	1.80%		8	3.83%	4	1.91%	
**Subtype**					***					0.894
HR+/HER2−	283	41.86%	88	39.64%		79	37.80%	84	40.19%	
HR+/HER2+	70	10.36%	37	16.67%		34	16.27%	37	17.70%	
HR−/HER2+	51	7.54%	25	11.26%		20	9.57%	22	10.53%	
HR−/HER2−	99	14.64%	45	20.27%		47	22.49%	40	19.14%	
unknown	173	25.59%	27	12.16%		29	13.88%	26	12.44%	
**Race**					0.303					0.112
white	555	82.10%	171	77.03%		149	71.29%	161	77.03%	
black	68	10.06%	26	11.71%		37	17.70%	25	11.96%	
other	49	7.25%	24	10.81%		20	9.57%	23	11.00%	
unknown	4	0.59%	1	0.45%		3	1.44%	0	0.00%	
**Histological type**					***					0.671
IDC	165	24.41%	109	49.10%		91	43.54%	100	47.85%	
ILC	71	10.50%	5	2.25%		5	2.39%	5	2.39%	
other	440	65.09%	108	48.65%		113	54.07%	104	49.76%	
**Marital status**					0.075					0.995
married	322	47.63%	128	57.66%		118	56.46%	119	56.94%	
divorcedsSeparated/other	111	16.42%	30	13.51%		27	12.92%	28	13.40%	
single	102	15.09%	32	14.41%		31	14.83%	32	15.31%	
widowed	119	17.60%	25	11.26%		27	12.92%	24	11.48%	
unknown	22	3.25%	7	3.15%		6	2.87%	6	2.87%	
**N stage**					***					0.688
N0	166	24.56%	6	2.70%		4	1.91%	6	2.87%	
N1	331	48.96%	145	65.32%		144	68.90%	136	65.07%	
N2	38	5.62%	37	16.67%		26	12.44%	33	15.79%	
N3	70	10.36%	34	15.32%		35	16.75%	34	16.27%	
unknown	71	10.50%	0	0.00%		0	0.00%	0	0.00%	
**Grade**					***					0.321
well	2	0.30%	4	1.80%		2	0.96%	4	1.91%	
moderately	15	2.22%	13	5.86%		6	2.87%	12	5.74%	
poorly	71	10.50%	56	25.23%		43	20.57%	48	22.97%	
unknown	588	86.98%	149	67.12%		158	75.60%	145	69.38%	
**Median household income (inflation adjusted)**					0.659					0.437
<45,000$	49	7.25%	19	8.56%		14	6.70%	18	8.61%	
45,000–54,999$	87	12.87%	34	15.32%		21	10.05%	32	15.31%	
55,000–64,999$	159	23.52%	55	24.77%		53	25.36%	53	25.36%	
65,000–74,999$	157	23.22%	51	22.97%		54	25.84%	47	22.49%	
>74,999$	224	33.14%	63	28.38%		67	32.06%	59	28.23%	
**Chemothrapy**					***					0.466
no/unknown	295	43.64%	24	10.81%		30	14.35%	24	11.48%	
yes	381	56.36%	198	89.19%		179	85.65%	185	88.52%	
**Radiotherapy**					***					0.768
no/unknown	407	60.21%	96	43.24%		90	43.06%	94	44.98%	
yes	269	39.79%	126	56.76%		119	56.94%	115	55.02%	
**Bone metastases**					***					1
no	393	58.14%	216	97.30%		205	98.09%	203	97.13%	
yes	265	39.20%	3	1.35%		1	0.48%	3	1.44%	
unknown	18	2.66%	3	1.35%		3	1.44%	3	1.44%	
**Liver metastases**					***					0.604
no	576	85.21%	218	98.20%		206	98.56%	205	98.09%	
yes	79	11.69%	1	0.45%		1	0.48%	1	0.48%	
unknown	21	3.11%	3	1.35%		3	1.44%	3	1.44%	
**Lung metastases**					***					0.6
no	568	84.02%	216	97.30%		200	95.69%	203	97.13%	
yes	86	12.72%	3	1.35%		6	2.87%	3	1.44%	
unknown	22	3.25%	3	1.35%		3	1.44%	3	1.44%	
**Brain metastases**					**					0.784
no	617	91.27%	216	97.30%		205	98.09%	203	97.13%	
yes	37	5.47%	2	0.90%		1	0.48%	2	0.96%	
unknown	22	3.25%	4	1.80%		3	1.44%	4	1.91%	

** *p* < 0.01, *** *p* < 0.001.

## Data Availability

All data here are publicly available in the SEER database (https://seer.cancer.gov/ (accessed on 15 April 2022)).

## Supplementary Materials

The following supporting information can be downloaded at: https://www.mdpi.com/article/10.3390/jcm12093097/s1, Table S1: Baseline characteristics of occult breast cancer (OBC) patients in train set and test set; Table S2: Univariate and multivariate Cox analysis of OBC characteristics extracted from train data; Table S3: Univariate and multivariate Cox analysis of OBC characteristics (stratified by molecular subtype).
